# Predictive Factors for Procedure Time for Closure of Mucosal Defect Following Colorectal Endoscopic Submucosal Dissection

**DOI:** 10.1002/jgh3.70174

**Published:** 2025-05-07

**Authors:** Hideaki Kazumori, Rurika Masatsugu, Kousuke Fukuda, Koji Onishi, Yasuhiko Ohno

**Affiliations:** ^1^ Department of Gastroenterology Matsue Seikyo General Hospital Matsue Shimane Japan

**Keywords:** clip closure method, colorectal neoplasm, loop clip closure method, multiple linear regression model

## Abstract

**Goals:**

The present study was conducted to clarify predictive factors related to procedure time for closure of a mucosal defect following colorectal endoscopic submucosal dissection.

**Background:**

To prevent complications following a colorectal endoscopic submucosal dissection (ESD) procedure, closure of the resultant mucosal defect is considered to be most effective. However, closure after colorectal ESD is challenging, and technical difficulties can lead to a longer procedure time. Although it is important to clarify predictive factors related to the time needed for effective treatment planning, no such validated data obtained prior to the present study have been reported.

**Study:**

Overall, 61 consecutive patients who underwent colorectal ESD for a colorectal neoplasm sized greater than 20 mm were enrolled. Immediately after performing colorectal ESD, closure of the mucosal defect was implemented using a loop clip closure method. Factors with influence on closure procedure time were evaluated using multiple linear regression analyses.

**Results:**

Results obtained with a multiple linear regression model demonstrated that resected specimen size (*β* = 0.690, *p* < 0.01) and colon site (*β* = −0.209, *p* = 0.027) were factors with influence on the closure procedure. Those results were considered relevant to explain the 50.5% variance in time until completion of closure; thus, goodness of fit was considered to be high.

**Conclusions:**

Findings obtained in this study were helpful to clarify predictive factors with influence on procedure time. The fit of the model was good, thus allowing for closure performance based on outcome prediction.

**Abbreviation**
ESD: endoscopic submucosal dissection

## 1 Introduction

Endoscopic submucosal dissection (ESD) has become a widely accepted treatment method and is increasingly being used for colorectal neoplasm cases [1], with low local rates of recurrence and high‐quality pathological specimens for accurate histological diagnosis obtained [2]. While large lesions can be resected en bloc, resultant mucosal defects are also large, which can lead to adverse events, particularly delayed bleeding or perforation, or post‐electrocoagulation syndrome [3, 4]. To prevent such occurrences, prophylactic closure of the mucosal defect is considered to be most effective, as the majority of studies that examined the effectiveness of closure have noted its usefulness for the prevention of adverse events [5, 6, 7, 8, 9], while a few reports have presented results showing no significant relationship of decreased incidence of complications with prophylactic endoscopic closure following a colorectal ESD procedure [10, 11].

A large mucosal defect beyond the range of the clip arms cannot be easily closed using a conventional standard clip closure method; thus, several techniques have been developed for such a condition [5, 6, 12, 13, 14, 15, 16, 17, 18, 19, 20, 21, 22, 23]. Nevertheless, those studies showed that closure for a mucosal defect following a colorectal ESD procedure can be challenging, with the rate of success ranging from 73% to 100%. Notably, closure must be completed using a technique associated with a high rate of success. Should delayed bleeding occur, hemostasis can be difficult because of interference by the clip, resulting in an incomplete procedure.

Because of the variety of mucosal defect conditions encountered and closure methods available, it is difficult to make a general conclusion, though some difficult cases require a long time for closure. When an extended period is required to perform ESD, the general condition of the patient may deteriorate because of the additional time used for closure. Thus, clarification of predictive factors affecting closure time is of great value for arranging the procedure schedule and improving the quality of closure outcomes. Generally, it is difficult to predict procedure time in cases being treated for lesions with large mucosal defects, while it has also not been clarified whether procedure time increases only in association with increased size. Even in the current environment with several good methods being developed, no known report concerning factors related to the prediction of procedure time for closure following colorectal ESD has been presented.

For the present patients, closure of mucosal defects was executed following colorectal ESD using a previously reported loop clip closure method [24], with minor modifications. Analysis was then performed to clarify predictive factors that influenced the closure plan.

## 2 Patients and Methods

A total of 61 consecutive patients who underwent colorectal ESD treatment for colorectal neoplasms between May 2019 and February 2020 at Matsue Seikyo General Hospital were enrolled in this study. All ESD and mucosal defect closure procedures were performed by three experienced endoscopists at our institution, who have each performed more than 100 colorectal ESD and mucosal defect closure procedures. All of the patients had resection specimens larger than 20 mm, and closure of the mucosal defect was performed immediately after ESD. Procedure time for closure was defined as the period from preparation of the first loop‐clip to attachment of the last clip and included preparation of clips, scope repositioning, and/or troubleshooting failed clip placement. Closure time, success rate of complete closure, and complications were retrospectively analyzed, while factors with an influence on procedure time for closure were also determined. The location of the flexural area was determined based on hepatic and splenic flexure, and the sigmoid descending and rectosigmoid junctions.

### 2.1 Clip Closure Method

All procedures were performed with a single channel using the same scope as used for ESD. The loop clip closure method for the present study was the same as previously reported [24], with modifications (Video S1). Briefly, a clip (ZEOCLIP, ZP‐CH; Zeon Medical INC., Tokyo, Japan) is prepared with a 3–0 nylon thread tied onto the hole of the clip arm and a 7 mm in diameter loop made with the nylon thread. The clip with the nylon thread loop is used to grasp one side of the edge of the mucosal defect. A second clip device without a nylon loop is then passed through the nylon loop of the first clip and rotated so as to be wrapped with the nylon loop. Next, after dragging the second clip along with the first clip to the other side of the mucosa, the second clip is attached to the mucosal edge. A third clip (HX‐202UR, Olympus Corp., Tokyo, Japan) is attached to the ends of the roots of both the first and second clips. Additional clips are then attached next to the third clip in sequence, and closure is completed in a zipper manner. For the present analysis, complete closure was defined as complete suturing of the defect for the entire resection site in a zipper manner, while incomplete closure was defined as a mucosal defect only partially sutured.

### 2.2 Statistical Analysis

Values are presented as the mean ± SD. Normality of variables was examined using the Shapiro–Wilk test. Continuous variables among multiple samples regarding colon site were compared using a Kruskal–Wallis test for variables with non‐normal distributions.

Pearson's correlation coefficient and simple linear regression models were used to assess the relationship between procedure time for closure and independent variables. Stepwise multiple linear regression was conducted to build a candidate model. *p*‐values less than 0.05 were considered to be statistically significant. Statistical analyses were performed using the SPSS software package, version 29.0.

## 3 Results

**FIGURE 1:**
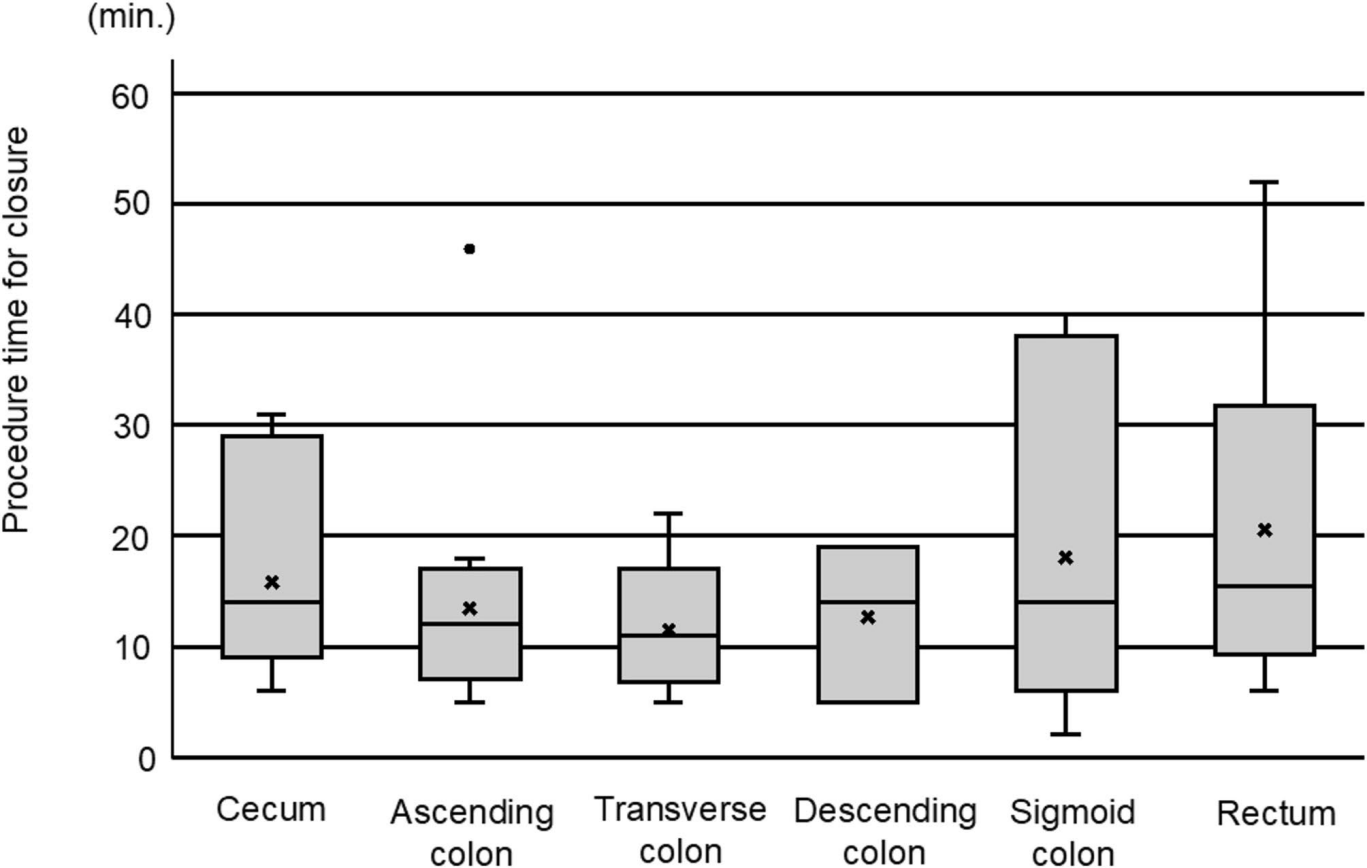
Procedure times for closure of mucosal defect after endoscopic submucosal dissection among colon sites, including cecum, ascending colon, transverse colon, descending colon, sigmoid colon, and rectum. There were no significant differences for required closure time among the sites.

Characteristics of the 61 enrolled patients and closure outcomes are summarized in Table 1. Complete mucosal defect closure was achieved in 59 (96.7%) of the patients, while failure was noted in two (3.3%). Those 59 cases with successful closure were included in the following analyses. The time for the clip closure procedure was 15.0 ± 10.6 min. None of the patients developed delayed bleeding or perforation. There were no significant differences regarding the time required for closure among the colon sites in the present cases (Figure 1).

**TABLE 1:** Patient characteristics and closure outcomes.

Patient characteristics	(*n* = 61)
Age, years (mean ± SD)	68.1 ± 13.6
Male/female	31 (50.8%)/30 (49.2%)
Colon site	
Cecum/ascending/transverse/sum	12/16 / 14/42 (68.9%)
Descending/sigmoid/rectum/sum	3/8 / 8/19 (31.1%)
Location of flexural area	
Flexural area	16 (26.2%)
Non‐flexural area	45 (73.8%)
ESD factors	
En bloc resection	61 (100%)
Procedure time for ESD, min. (mean ± SD)	62.1 ± 63.9
Size of resected specimen, mm. (mean ± SD)	34.3 ± 13.8
Pathological diagnosis	
Adenoma	50 (82.0%)
Adenocarcinoma (intramucosal/submucosal)	7 (11.5%)/4 (6.6%)
Complication associated with ESD	
Bleeding	0 (0%)
Perforation	0 (0%)
Closure outcome	
Complete closure	59 (96.7%)
Incomplete closure	2 (3.3%)
Procedure time for complete closure, min. (mean ± SD)	14.9 ± 10.6
Complications associated with complete closure	
Delayed peritonitis	1 (1.7%)
Delayed bleeding	0 (0%)
Delayed perforation	0 (0%)
Number of loop clips required for complete closure	
1	49 (83.1%)
2	8 (13.6%)
3	2 (3.4%)

*Note:* Values show number, unless otherwise indicated.

Abbreviation: ESD: endoscopic submucosal dissection.

### 3.1 Correlation Coefficient Results

Pearson's correlation coefficient calculations were performed for each pair of dependent and independent variables, with the results presented in Table 2. Age, gender, colon site, location in flexural area, size of resected specimen, and procedure time for ESD were included in the analysis. The results showed a significant association of procedure time for closure with size of the resected specimen [Pearson's correlation coefficient (*r*) =0.691, *p* = 0.000] and ESD procedure time (*r* = 0.369, *p* = 0.002). In addition, a positive correlation between resected specimen size and the number of loop clips required for closure was noted (*r* = 0.428, *p* < 0.001).

**TABLE 2:** Pearson's correlation coefficient matrix.

	Procedure time for closure	Age	Gender	Colon site	Location of flexural area	Size of resected specimen
Age	0.079 (0.276)					
Gender	0.101 (0.223)	0.106 (0.212)				
Colon site	−0.213 (0.053)	0.171 (0.097)	0.187 (0.078)			
Location of flexural area	0.104 (0.216)	−0.208 (0.057)	−0.045 (0.367)	−0.175 (0.092)		
Size of resected specimen	0.691 (0.000)	0.122 (0.179)	0.185 (0.080)	−0.005 (0.486)	−0.018 (0.447)	
Procedure time for ESD	0.369 (0.002)	0.037 (0.390)	0.086 (0.259)	−0.147 (0.133)	0.089 (0.252)	0.691 (< 0.001)

*Note:* Numbers in parentheses indicate *p* values.

Abbreviation: ESD: endoscopic submucosal dissection.

### 3.2 Simple Linear Regression Analysis

Results of factors subjected to simple linear regression analysis, including age, gender, colon site, location in flexural area, size of resected specimen, and procedure time for ESD, are shown in Table 3. It was found that closure time could be predicted based on resected specimen size (*β* = −0.691, *p* < 0.001) and ESD procedure time (*β* = −0.369, *p* = 0.004). In addition, closure was shown to account for 47.8% and ESD procedure time for 13.6% of the variance in time required to complete closure in the analyzed cases.

**TABLE 3:** Simple linear regression analysis for procedure time of closure of mucosal defect.

	Factor values	*R* ^2^	*β*	*p*	95% CI
Age	Primary value	0.006	0.079	0.551	−0.1417—0.273
Gender	0 = Male, 1 = Female	0.010	0.101	0.446	−3.440—7.712
Colon site	0 = D, S, R; 1 = C, A, T	0.045	−0.213	0.106	−10.809—1.069
Location of flexural area	0 = No, 1 = Yes	0.011	0.104	0.433	−3.790—8.732
Size of resected specimen	Primary value	0.478	0.691	< 0.001	0.429–0.757
Procedure time for ESD	Primary value	0.136	0.369	0.004	0.020–0.102

Abbreviations: A; ascending colon, C: cecum, CI: confidence interval, D: descending colon, ESD: endoscopic submucosal dissection, R: rectum, *R* ^2^: coefficient of determination, S: sigmoid colon, T: transverse colon, *β*: standardized partial regression coefficient.

### 3.3 Stepwise Multiple Linear Regression Analysis

The results of stepwise multiple linear regression analyses, performed for determining the relationships of procedure time for closure with age, gender, colon site, location of flexural area, size of resected specimen, and procedure time for ESD, are shown in Table 4. The final multiple linear regression model demonstrated that resected specimen size (*β* = 0.690, *p* < 0.01) and site in the colon (*β* = −0.209, *p* = 0.027) were factors with influence on closure procedure time. Furthermore, the analysis of variance results was significant and indicated that the model could explain the 50.5% variance in time until completion of closure [coefficient of determination (*R* ^2^) =0.522, adjusted *R* ^2^ = 0.505], the goodness of fit was considered to be high.

**TABLE 4:** Stepwise multiple linear regression analysis of procedure time for closure of mucosal defect.

	*B*	*β*	*p*	95.0% CI	VIF
Constant	−1.471		0.646	−7.854—4.911	
Size of resected specimen	0.592	0.690	< 0.01	0.433–0.751	1.000
Colon site	−4.794	−0.209	0.027	−9.037 to −0.552	1.000

Abbreviations: B: partial regression coefficient, CI: confidence interval, VIF: inflation factor, β: standardized partial regression coefficient.

Preliminary analysis using the Shapiro–Wilk test was performed to confirm normality for the quantitative variables. For analysis of the correlation matrix table, all variables were included, as none showed |*r*| > 0.8. All variance inflation factors were ≤ 10.0 and there were no problems with multicollinearity. The Durbin‐Watson ratio was 2.355, with no outliers considered to cause the predicted value to exceed±3SD relative to the actual value.

## 4 Discussion

Technical difficulties are generally the reason for longer procedure time in ESD cases. Similarly, the presence of a difficult‐to‐close mucosal defect following colorectal ESD may result in an extended period required to complete the procedure. For proper closure of a mucosal defect following colorectal ESD, it is important to consider factors with possible effects on procedure time. However, no data regarding the influence of such factors has been presented; thus, the present study was conducted to examine possible factors related to the time required for closure following a colorectal ESD.

Prophylactic clipping for a mucosal defect following a colorectal endoscopic resection procedure has been reported to be useful, especially for lesions greater than 20 mm in size [25, 26, 27]. The present study analyzed cases in which closure of a mucosal defect sized greater than 20 mm after a colorectal ESD was performed.

Among available endoscopic closure techniques, a previously reported loop clip closure method [24] was used for the present patients, with minor modifications. Various clip closure and similar methods with a ring or loop attached to the clip have been presented, each of which has various advantages [22, 28, 29, 30, 31]. A key benefit of the method used for the present cohort is that wrapping a thread around the second clip increases the gripping force of the first clip, while the use of the second along with the first clip makes it possible to draw or push the end of the mucosa to the desired site. Furthermore, thread wrapping makes the gap between the first and second clips smaller, and also shortens the distance between the target mucosa and the other side, thus making it easier to attach the third clip. With the use of this method, complete closure of the mucosal defect was achieved in the present cases with a high success rate of 96.7%.

In discussion regarding the present findings, colon site was thought to possibly have an impact on procedure time for closure. To use colon site as a dummy variable, the impact of specific sites on time for closure was examined, though none was found to have an effect on closure procedure time. Therefore, for consideration of endoscope maneuverability, the colon was divided into two sites: proximal, including the cecum and ascending and transverse colon, and distal, including the descending and sigmoid colon and rectum.

First, Pearson's correlation test and simple linear regression analysis were performed. Those results showed that both the size of the resected specimen and ESD procedure time had high correlations with closure time and were thus considered to be significant prediction values for the time of closure and used in a simple linear regression model. Results of the present analysis showed that the size of the resected specimen had a significant correlation with the time for the closure procedure, while a significant influence on the time for closure was also noted in simple regression model findings. Along with increased mucosal defect size, the number of clips required also increased, resulting in a longer procedure time, which may explain why that factor was shown to have influence in multiple regression model results.

Additionally, as the level of difficulty for performing ESD increases, the closure procedure is also expected to become more difficult. The present results showed that procedure time for ESD had a significant correlation with time for closure. However, a simple linear regression model indicated that the level of influence on closure time was not pronounced. Previous studies have noted that factors for determining colorectal ESD difficulty include tumor size, narrow lumen, site of colon, location of flexural area, endoscopic stability and maneuverability, and submucosal fibrosis, as well as others [32, 33, 34]. Additionally, one of the most important issues affecting colorectal ESD procedure time is the severity of fibrosis [35], though that has no technical relationship with closure, which may explain why it was not a factor showing influence in the present multiple regression model results.

Although endoscopic maneuverability in the flexural area has been suggested as an important factor for closure, it had no significant correlation with the time for the closure procedure in the present analysis. Furthermore, that showed only a weak level of influence on closure time in simple linear regression model findings. When performing the procedure for closure, there is no need for the endoscope to remain attached to the colon wall, as with ESD. When a large lesion is located in the flexural area, closure may not be easy; thus, it might be necessary to change the axis. However, once adequate shortening of the distance from the target mucosa to another part of the mucosa is successfully obtained by pulling on the loop clip at the site where the closure axis should be changed, the addition of more clips is the only requirement; thus, the presence or absence of a flexure area may not have an effect.

Age, gender, colon site, location of flexural area, size of resected specimen, and procedure time for ESD were factors analyzed, with size of resected specimen and colon site included in the multiple linear regression model. Resected specimen size had the largest βvalue and was considered to have the most influence, though colon site was also found to be an important factor with significant impact. Interestingly, colon site, whether proximal or distal, did not have a significant correlation with time for closure in simple linear regression model findings, whereas a significant influence on closure time was noted in multiple linear regression model findings. Two possible reasons were considered for the influence of colon site noted in the multiple regression model. First, for cases with a large defect, especially when located in a thick wall such as that of the rectum, gripping force is overcome by the pulling force of the loop clip, with detachment the result. Second, when a large mucosal defect is present in a narrow lumen such as the sigmoid colon, closure of only mucosa distant from the muscular layer will create a submucosal space. In such cases, the first loop clip should be attached in a location approximately one‐third of the distance from the edge, after which additional clips will be needed.

Although six factors were examined in this study, there may be others that should be considered. The experience of the endoscopist is a key predictive factor for a difficult procedure. Indeed, the rate of perforation in colorectal ESD patients was shown to significantly decrease after experience with 40 cases, while proficiency with the procedure has been shown to prevent complications [36]. Data analyzed in the study were obtained only from cases treated by skilled operators. It is considered necessary to stratify endoscopists based on their skills for the closure procedure. Nevertheless, patient factors should also be considered, since it is known that the perforation rate associated with ESD is reduced in obese patients with a body mass index > 30 kg/m^2^, likely due to the thicker colon wall in such cases, though direct evidence has yet to be presented [37]. For closure of a thick wall, the pulling force of the loop clip overpowers the gripping force, likely making it easier to become detached; thus, a longer procedure time will be necessary. The presence of diverticula is also a risk factor for perforation; thus, ESD was previously generally contraindicated for diverticulum‐associated colorectal lesions. Recently, increasing numbers of successful ESD for cases with diverticulum‐associated colorectal lesions have been reported; thus, the indications for ESD itself are expanding [38]. In future studies, it may be important to consider the existence of diverticula as a predictive factor.

Aside from the advantages and high success rates noted for this method, the principles are the same as with other clip closure methods, such as how to shorten the distance between the targeted and opposite side mucosa to facilitate additional placement of clips, and also sequence. Based on the method used, they can be considered as a single group, as clip‐based closure techniques, including technical skill requirements and procedure time, are considered to provide similar results [39]. The predictive factors are also applicable to other related methods, such as the use of a loop or ring attached to clip arms, as the principle is the same.

In addition to clarification of predictive factors, the present results may indicate a new tool that can be utilized clinically and is useful for predicting the time required for closure. The fit of the model was also shown to be good, with the final multiple regression model results indicating a variance in the time to completion of closure of 50.5%. Following ESD, procedure time can be predicted after measuring the resected specimen with the use of the following formula: Predicted procedure time (minutes) = −1.471 + 0.592 × size of resected specimen—4.794 × colon site. Clinically, for treatment planning it is sufficient to know the approximate time required; thus, estimation of the approximate size of the resected specimen in centimeters based on the condition of the lesion prior to ESD will provide adequate information needed for understanding the time required. The general condition of the patient and risk of complications can then be taken into account to consider whether closure should be performed.

The primary limitation of the present study is the use of results obtained at a single institution; thus, it is considered that the procedure time prediction formula can be used without modification only for cases treated by our department members. Establishment of a single formula for use by a variety of institutions is not possible, as endoscopic skills generally vary among practitioners, and there are also likely differences among facilities. Furthermore, since the number of cases in this study was small and the endoscopists who participated were limited to skilled operators, a common formula may be obtained by analyzing a greater number of cases treated at multiple institutions and stratifying them according to endoscopist skill level. Such results would be helpful to develop a treatment plan that takes into account not only ESD itself but also closure. Nevertheless, the two factors necessary for prediction are common, and it is considered that their intended use at the time of closure will assist with treatment planning.

## 5 Conclusions

Findings obtained in the present study showed that predictive factors for the time required for a mucosal defect closure after a colorectal ESD procedure are the size of the resected specimen and the colon site. These results are considered to provide important information for planning and details related to closure.

## Ethics Statement

The present study was performed in accordance with the Helsinki Declaration, and the protocol was reviewed and approved by the Institutional Review Board of Matsue Seikyo General Hospital (approval number 201905).

## Consent

Written informed consent for the endoscopic procedures and use of obtained data for scientific purposes was obtained from each of the patients.

## Conflicts of Interest

The authors declare no conflicts of interest. The authors have no relationship with industry related to this study.

## Supporting information


**Video S1.** Clip closure for mucosal defect after endoscopic submucosal dissection of specimen in transverse colon sized 50 × 40 mm.

## Data Availability

All data generated or analyzed during this study are included in the article. Further enquiries can be directed to the corresponding author.
